# Methodological proposal for validation of the disinfecting efficacy of an automated flexible endoscope reprocessor

**DOI:** 10.1590/1518-8345.0595.2745

**Published:** 2016-08-08

**Authors:** Kazuko Uchikawa Graziano, Marta Elisa Auler Pereira, Elaine Koda

**Affiliations:** 1 PhD, Full Professor, Escola de Enfermagem, Universidade de São Paulo, São Paulo, SP, Brazil.; 2 RN, Specialist in Occupational Health Nursing.; 3 RN, Specialist in Surgical Center.

**Keywords:** Methodology, Disinfection, Efficacy, Endoscopes

## Abstract

**Objective:**

to elaborate and apply a method to assess the efficacy of automated flexible endoscope reprocessors at a time when there is not an official method or trained laboratories to comply with the requirements described in specific standards for this type of health product in Brazil.

**Method:**

the present methodological study was developed based on the following theoretical references: International Organization for Standardization (ISO) standard ISO 15883-4/2008 and Brazilian Health Surveillance Agency (Agência Nacional de Vigilância Sanitária - ANVISA) Collegiate Board Resolution (Resolução de Diretoria Colegiada - RDC) no. 35/2010 and 15/2012. The proposed method was applied to a commercially available device using a high-level 0.2% peracetic acid-based disinfectant.

**Results:**

the proposed method of assessment was found to be robust when the recommendations made in the relevant legislation were incorporated with some adjustments to ensure their feasibility. Application of the proposed method provided evidence of the efficacy of the tested equipment for the high-level disinfection of endoscopes.

**Conclusion:**

the proposed method may serve as a reference for the assessment of flexible endoscope reprocessors, thereby providing solid ground for the purchase of this category of health products.

## Introduction

In Brazil, requests to register electromedical equipment according to Health Surveillance standards should meet (among others) the requirements described in the Brazilian Health Surveillance Agency (Agência Nacional de Vigilância Sanitária -ANVISA) Collegiate Board Resolution (Resolução de Diretoria Colegiada - RDC) no.56/2001^1^, which "Establishes the essential safety and efficacy standards applicable to health products". Proof that these standards are met must be demonstrated through (but not limited to) equipment conformity certification according to the Brazilian System of Conformity Assessment. 

The conformity assessment of health products first targeted electromedical equipment with the publication of the Health Ministry/Health Surveillance Secretary Ordinance no. 2,663 on December 22, 1995, and is currently regulated by ANVISA RDC no. 27/2011^2^. During that period of time, ANVISA delivered several related publications to make the procedures and deadlines indicated in the initial ordinance compatible with the market's ability to meet them while product certification laboratories were being trained and accredited by the National Institute of Metrology, Quality and Technology (Instituto Nacional de Metrologia, Qualidade e Tecnologia - INMETRO) to perform the corresponding assays^3^_._

For users of Brazilian health services (i.e., health care providers and patients alike), mandatory certification of electromedical equipment represents an important advance that ensures the quality of the products available in the market based on their safety and efficacy and consequently patient safety. In other words, consumers purchasing electromedical equipment are granted the right of access to confirmatory data demonstrating that a given device truly performs its functions in a satisfactory manner, thereby allowing the attainment of the intended results. 

However, a robust official method based on theoretical frameworks must be elaborated before electromedical equipment certification laboratories can be trained. The method used in the present study arose from the need to demonstrate the safety and efficacy of an automated endoscope reprocessor at a time when there was not an official method available that met specific standards, such as the Brazilian National Standards Organization (Associação Brasileira de Normas Técnicas - ABNT) Brazilian Standard (Norma Brasileira - NBR) ISO 15883-1:2013 and international standard ISO 15883-4:2008, which apply to this type of health product. 

Flexible endoscopes are complex instruments that are introduced into the human body and thus become contaminated during the course of their use in clinical routines. Because this type of electromedical equipment does not usually come in contact with sterile tissue, it is rated as semi-critical^4^ ^-^ ^5^. Therefore, flexible endoscopes should at least be subjected to minimum reprocessing between one patient and the next, which includes cleaning and high-level disinfection via the manual or automated method to prevent cross-contamination between patients^6^. 

The proposal and assessment of the applicability of a robust model for the analysis of the efficacy of automated flexible endoscope reprocessors that will be launched onto the market in accordance with the legal provisions account for the relevance of the present study. Thus, this study fills a gap in the current knowledge. 

The objective of the present study was to develop a method to assess the efficacy of automated flexible endoscope reprocessors and to analyze the method's feasibility and the results obtained by application to a specific brand and model. 

## Methods

The present methodological study was elaborated and conducted in the city of São Paulo, São Paulo, Brazil, in 2014. The following official documents were considered in the elaboration of the assessment method: ISO 15883-4/2008, RDC no. 35/2010^7^ ^)^ and RDC no. 15/2012^8^. 

**Figure 1:**
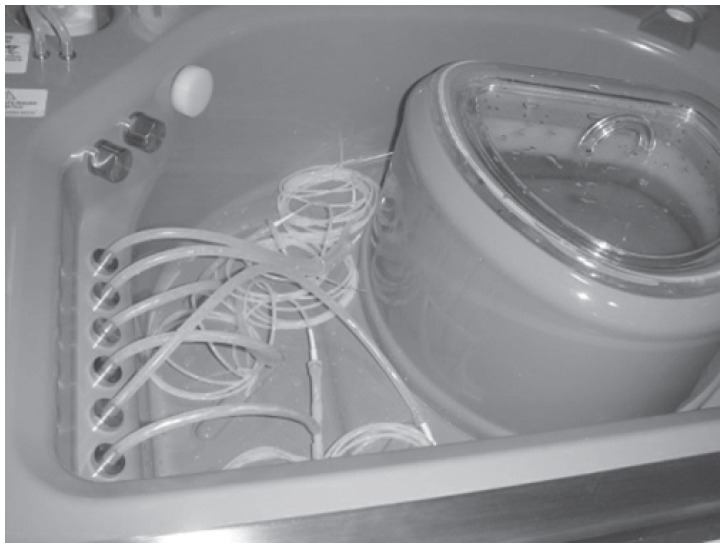
Test specimens fitted into the connectors of the tested automated endoscope reprocessor.

The test specimens were new and translucent polytetrafluoroethylene (Teflon(r)) tubes with a 1,500 mm length (as indicated by ISO 15883-4) and 1.0 mm internal diameter (the diameter of the smallest endoscope channel (air or water channel) was selected as the worst-case scenario). The material was purchased from a company accredited by its manufacturer (Dupont(r)) to market this product, which was similar to the raw material included in endoscope channels as demonstrated by a Brazilian study^9^. The test specimens were directly fitted into the tested device connectors as shown in Figure 1. 

The proposed method was applied to a device made in Brazil that was indicated for automated reprocessing of the primary locally available flexible endoscope brands. The reprocessor is used for high-level disinfection and allows programing of the following steps: leak testing, detergent flushing (with or without enzymes) and rinsing, high-level disinfection and rinsing, and drying of the endoscope channels. 

During high-level disinfection, the endoscope is fully immersed in the disinfecting solution, which promotes the passing of the latter through the channels for the amount of time established by the disinfectant manufacturer^10^. This step may be programmed to last up to 60 minutes. 

The device requests and saves the corresponding date of the first use of a disinfectant bottle that is expected to be reused according to the manufacturer's instructions. These data are informed at the onset of each operational cycle. 

At the end of the high-level disinfection step, the disinfecting solution returns to a specific storage compartment in the device, where it remains until it is reused. The concentration of the disinfecting solution should be checked at least once daily in compliance with RDC no. 15/2012^8^.

The rinsing step begins automatically following high-level disinfection; the endoscope is rinsed with purified water (passed through a 5-µm filter) to remove all disinfectant residues from both the endoscope channels and external surfaces. Air is passed through the channels at each rinsing stage and also at the end of the process to drain the water used for rinsing. 

To validate the high-level disinfection efficacy of the tested reprocessor, a disinfectant containing 0.2%* peracetic acid was selected as the active principle. The process was programmed as follows: contact with disinfectant for 10 minutes, followed by two full rinsing stages with purified water, and passing of air through the channels for 1 minute. This sequence represents the basic cycle. 

The test specimens were intentionally contaminated with challenge microorganisms. The microorganisms listed in RDC no. 35/2010^7^ and ISO 15883-4/2008 for high-level disinfectant assessment were selected as follows: *Staphylococcus aureus (*ATCC* 6538*), Pseudomonas aeruginosa* (ATCC 15442), *Candida albicans (*ATCC 10231*), Mycobacterium massiliense* (INCQS† 00594), and *Bacillus subtilis/atropheus (*ATCC 6633*).* The latter bacterium was included in its sporulated form. 

### Step-by-step description of the proposed method

A 25-µl aliquot of the culture medium was placed into each test specimen using an automatic pipette. The specimens were rotated until their lumens became visibly dry. This procedure was repeated three times following ISO 15883-4.

The contaminated test specimens were exposed to the disinfection cycle using the chosen disinfectant at two time points as follows: when the disinfectant condition was best (i.e., its first two uses) and when it was worst (i.e., the 51^st^ and 52^nd^ reuses). The mean of the maximum number of reuses guarantees the presence of the required active principle concentration. Disinfectant reuse was simulated by setting the device in the absence of test specimens. During the experiments, the concentration of peracetic acid was measured in duplicate during each cycle using a validated colorimetric test strip to monitor the curve of decay of the active principle as a function of the number of reuses. 

The culture method used to quantify the number of surviving microorganisms after exposure to the disinfectant was previously validated and shown to be capable of recovering a low number of microorganisms (approximately 10), which complied with standard ISO 15883-4.

To neutralize the peracetic acid, a 0.01 mol.L^-1^ or 0.4 g.L^-1^ NaOH solution was added to the culture medium.

### Study groups and sample size

Standard ISO 15883-4 recommends performing all tests in duplicate. Thus, the sample size was defined by the groups described below. 

Experimental group: two test specimens per tested microorganism; 5 test specimens were subjected to the disinfectant in its first use, 5 to its first reuse, 5 to its 50^th^ reuse, and 5 to its 51^st^ reuse for a total n=20. This allocation was necessary because the equipment did not allow exposure to all of the test specimens at once. 

Positive control group: comprised non-reprocessed test specimens (n=2 per tested microorganism) contaminated with the 5 tested microorganisms for a total n=10. 

Negative control group: new, clean, and sterilized test specimens not subjected to intentional contamination for a total n=2. 

### Methods for the quantitative recovery of microorganisms from the experimental group in compliance with standard ISO 15883-4

The test specimens were cut into four cross-sectional segments using an aseptic technique. Each cannulated segment was opened lengthwise using a sterilized scalpel. Next, two segments from each specimen were transferred to a sterilized glass container with a screw cap containing 20 mL of sterile 1/4 strength Ringer solution containing 0.05% polysorbate 80.

The container was subjected to an ultrasonic bath 3 times at 45 kHz for 5 seconds. Next, the container was agitated by orbital motion for 10 minutes. The eluate was used to prepare a series of dilutions that were used to count the viable microorganisms. The other two segments from each specimen were used in the qualitative microbial recovery tests using adequate culture media (growth/non-growth assay). 

### Control groups

The same procedure was used for the positive controls. Following intentional contamination, the specimens were subjected to the microbial recovery tests without having previously undergone high-level disinfection in the tested equipment.

Similarly, the negative controls were subjected to the same microbial recovery testing procedures. 

### Interpretation of the results

The results were analyzed based on the change of each microbial population expressed as log_10_ as indicated in section 4.4.2.4 of standard ISO 15883-4. A device was considered effective when at least 6 logs of vegetative bacteria, fungi, and yeasts, at least 5 logs of mycobacteria, and at least 4 logs of fungal spores and viruses were inactivated. 

## Results

According to the results, the equipment maintained its efficacy for the high-level disinfection of endoscopes for the tested microorganisms after the 51^st^ cycle using the selected disinfectant with an exposure time of 10 minutes, followed by two rinsing stages and passage of air through the channels for 1 minute (Table 1). 

**Table 1:** Results of the validation analysis of disinfection efficacy. São Paulo-SP, Brazil, 2014.

Tested microorganism	Microorganism count in test specimens used as positive controls	Viable microorganism count in test specimens after reprocessing cycle 51
*Staphylococcus aureus* (ATCC 6538)	2 x10^6^ CFU^*^/ 37.5 cm test specimen	Absence/ 37.5 cm test specimen
*Pseudomonas aeruginosa* (ATCC 15442)	5x10^6^ CFU/ 37.5 cm test specimen	Absence/ 37.5 cm test specimen
*B. subtilis (ATCC 19659)*	3x10^4^ CFU/ 37.5 cm test specimen	Absence/ 37.5 cm test specimen
*M. massiliense* (INCQS #00594)	5x10^6^ CFU/ 37.5 cm test specimen	Absence/ 37.5 cm test specimen
*Candida albicans* (ATCC 10231)	1x10^6^ CFU/ 37.5 cm test specimen	Absence/ 37.5 cm test specimen

*CFU = colony forming unit

## Discussion

According to the DATASUS database, approximately 1.8 million procedures involving the use of flexible endoscopes were performed from March 2013 to April 2014 in Brazil. 

In a recent publication by the Emergency Care Research Institute (ECRI), cross-contamination from flexible endoscopes ranked sixth among the "Top 10" health technology hazards^11^.

Cases of infection associated with gastrointestinal endoscopy used to be rare and the few reported in the literature were attributed to errors in the performance of the standard endoscope reprocessing procedures or equipment failure^6^ ^,^ ^12^ ^-^ ^13^. Unfortunately, recent reports changed that scenario concerning not only the frequency but also the severity of the infection. In February 2015, the Ronald Reagan Medical Center at the University of California, Los Angeles, reported to the U.S. Food and Drug Administration (FDA) the occurrence of two deaths due to infection with carbapenem-resistant enterobacteriaceae in association with the performance of endoscopic retrograde cholangiopancreatography (ERCP); an additional five cases of infection by the same genus of multidrug-resistant bacteria were detected and possible exposures were identified in an additional 179 patients^14^.

From January 2013 through December 2014, the FDA received 75 notifications encompassing 135 cases in the United States related to possible microbial transmission, including multidrug-resistant bacteria, from reprocessed duodenoscopes (distal end)^15^. 

As a function of the aforementioned outbreaks, the various involved sectors (regulatory, standard setting, manufacturers, and specialized associations) began to search for new procedures and to develop projects aimed at achieving adequate endoscope reprocessing. 

While the market awaits these "new products", the best protection against cross-contamination from flexible endoscopes remains adequate reprocessing. This process involves rigorous cleaning and disinfection of endoscopes after use in patients according to the procedures recommended for this type of equipment^12^ together with the standard best practices for the prevention of healthcare-associated infections^6^.

Automated endoscope reprocessors were projected to standardize and automate manual reprocessing because this type of reprocessing was not always performed in an effective or consistent manner due to human errors, the large number of complicated stages required, and the pressure of services to reprocess endoscopes quickly between one patient and the next^4^.

The main advantages of automated endoscope reprocessors emphasized in the literature are as follows: standardization of the reprocessing steps with a reduction of the risk of human errors^16^, decreased odds of omitting an essential step^16^, direct contact of all the internal and external components and the lumens of the devices with the high-level disinfectant, uniform and reliable rinsing^16^, a reduction of the occupational exposure to disinfectants^4^ ^,^ ^17^, and decreased environmental contamination^16^.

All equipment involved in material reprocessing requires preventive maintenance^5^ and systematized monitoring of their performances. Concerning automated endoscope disinfection devices, there are reports of outbreaks of infection or colonization related to possible flaws in the water filtration system and the cleaning of endoscope channels and accessories^6^. Therefore, measures to prevent deviations in the quality of the expected performance, such as disinfectant dilution, contamination of water and air filters and low flow at the connectors exits, are crucial^18^ ^-^ ^19^.

Endoscopy associations call attention to the importance of adequate practices for equipment decontamination, bacteriological surveillance^20^, and monitoring of warning publications (by manufacturers or health surveillance authorities and the scientific literature) on automated endoscope reprocessor flaws that might result in infection.

The present pioneering study elaborated the first proposal to register this category of equipment with ANVISA and provided evidence of the efficacy and safety of an automated endoscope reprocessor. This result is particularly significant considering that no laboratories in Brazil are trained to analyze the safety of this type of equipment in conformity with the corresponding technical standards. 

**Figure 2:**
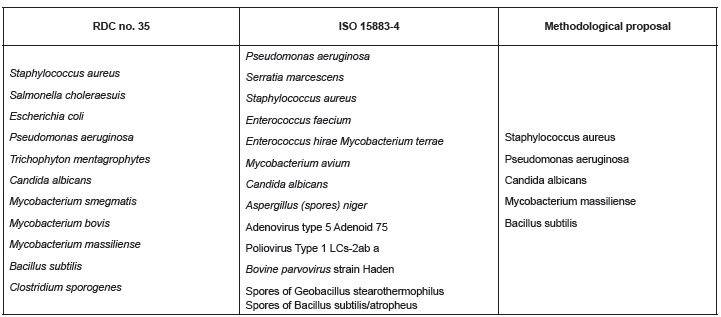
List of test microorganisms in RDC no. 35/2010(7) and ISO 15883-4 selected for the present methodological proposal.

During the elaboration of the protocol described here, the need arose to redefine the list of microorganisms used for testing because none of the contacted laboratories affiliated with the Brazilian Network of Health Analytic Laboratories (Rede Brasileira de Laboratórios Analíticos em Saúde - REBLAS) worked with the viruses indicated in ISO 15883-4. The redefined list includes microorganisms mentioned in both reference documents cited below to make the assays feasible and at the same time meet the health surveillance requirements; therefore, it is worth observing that the disinfectant used was subjected to microbiological assessment and registered by ANVISA in compliance with RDC no. 35/2010^7^ (Figure 2).

**Figure 3:**
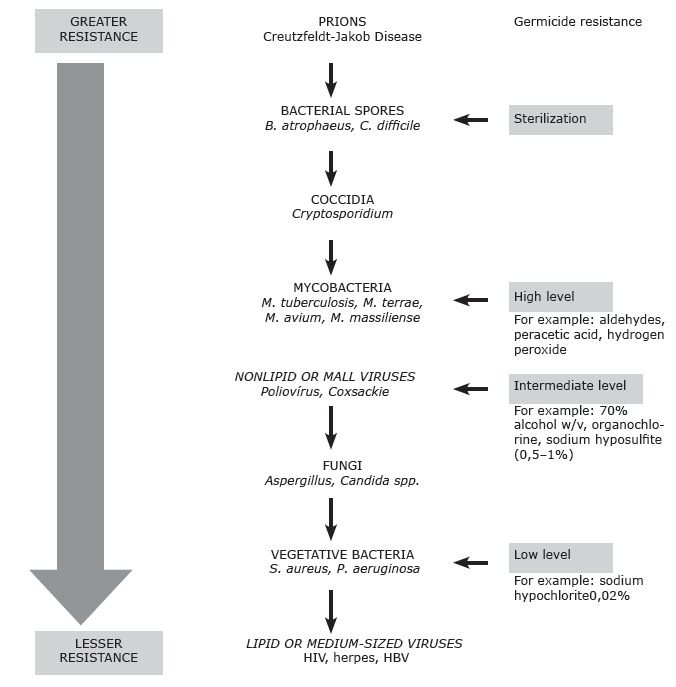
Decreasing order of resistance of microbial groups to chemical germicides according to the CDC(21)

The choice of test microorganisms for the present methodological proposal was scientifically based on the decreasing order of resistance of microbial groups to chemical germicides described by the Centers for Disease Control and Prevention (CDC)^21^ as shown in Figure 3

Therefore, the demonstration of efficacy against bacterial spores of *Bacillus subtilis* allows deductive inference that lipid viruses, including HBV (hepatitis B virus), HCV (hepatitis C virus), HIV (human immunodeficiency virus) and herpes, are also effectively eliminated. 

By comparison to the practical scenario studied in Brazil in 2011^16^ ^,^ ^19^ where endoscope contamination was primarily due to microorganisms belonging to the gut microbiota (*Escherichia coli, Klebsiella pneumoniae, Pseudomonas aeruginosa, Acinetobacter baumannii* and *Enterococcus faecalis*), we may infer that the microorganisms selected for the present methodological proposal represent a sufficiently challenging scenario. One possible explanation for the results of this field study, which recovered vegetative bacteria from the endoscope channel washing fluid, is the presence of dirt, which can prevent contact of the microorganisms with the chemical disinfectant. According to the decreasing order of resistance of microbial groups to chemical germicides described by the CDC^21^, the survival of vegetative bacteria suggests that the same situation may have occurred for the lipid viruses, which is regrettable from the perspective of patient (un)safety because this group includes HBV, HCV, and HIV. 

As a limitation of the present study, it was not possible to attain the concentration of 10^8^ recommended by the reference ISO standard for the inoculation of test microorganisms into test specimens during the preparation of the suspensions despite all of our efforts. However, the robustness of the assays was not impaired because all of the requirements for the assessment of the efficacy of disinfection using an automated flexible endoscope reprocessor indicated in the aforementioned standard were met, including the minimum logarithmic reduction of microorganisms required by ISO 15883-4. Another limiting factor is that although the material used represents a considerable challenging condition, it does not reproduce the physical shape and the challenges posed by the distal end of duodenoscopes. 

One additional aspect demonstrated in the present study was the maintenance of the minimum disinfectant concentration within the range specified by the manufacturer up to the 51^st^ reuse. This finding demonstrates that small, non-controllable dilutions of this chemical during its various reuses in the tested equipment do not interfere with its efficacy. 

## Conclusion

The proposed method was found to be feasible and reliable for the challenge imposed and could serve as a model for the assessment of similar devices and help healthcare professionals in the purchase of this category of health products. 

Considering the theoretical and methodological frameworks that grounded the present study, the tested equipment demonstrated efficacy and safety for use in clinical practice.
